# Development of biomaterial scaffold for nerve tissue engineering: Biomaterial mediated neural regeneration

**DOI:** 10.1186/1423-0127-16-108

**Published:** 2009-11-25

**Authors:** Anuradha Subramanian, Uma Maheswari Krishnan, Swaminathan Sethuraman

**Affiliations:** 1 Center for Nanotechnology & Advanced Biomaterials, School of Chemical & Biotechnology, SASTRA University, Thanjavur, India

## Abstract

Neural tissue repair and regeneration strategies have received a great deal of attention because it directly affects the quality of the patient's life. There are many scientific challenges to regenerate nerve while using conventional autologous nerve grafts and from the newly developed therapeutic strategies for the reconstruction of damaged nerves. Recent advancements in nerve regeneration have involved the application of tissue engineering principles and this has evolved a new perspective to neural therapy. The success of neural tissue engineering is mainly based on the regulation of cell behavior and tissue progression through the development of a synthetic scaffold that is analogous to the natural extracellular matrix and can support three-dimensional cell cultures. As the natural extracellular matrix provides an ideal environment for topographical, electrical and chemical cues to the adhesion and proliferation of neural cells, there exists a need to develop a synthetic scaffold that would be biocompatible, immunologically inert, conducting, biodegradable, and infection-resistant biomaterial to support neurite outgrowth. This review outlines the rationale for effective neural tissue engineering through the use of suitable biomaterials and scaffolding techniques for fabrication of a construct that would allow the neurons to adhere, proliferate and eventually form nerves.

## Introduction

The human brain is analogous to a black box of information and unraveling its mysteries is essential to understand its complex relationship with the various components of the peripheral and central nervous systems. This information is vital to probe the causes for various neural disorders and arrive at a plausible therapy for the treatment of ischemic, metabolic, congenital, or degenerative disorders of the central or peripheral nervous systems. Conventionally, autologous grafts are gold standards and have been used to treat neural defects [1-3]. However, autografts have limitations that include shortage of nerves since it is taken from the patient. Moreover, there is a mismatch of donor-site nerve size with the recipient site, neuroma formation and lack of functional recovery [4,5]. Allogenic grafts, which are isolated from cadavers, are not limited by supply but suffer from host-graft immune rejection [6]. To overcome immune rejection, several studies have been conducted to examine the potency of acellular nerve grafts [7,8]. However, as acellular nerve graft lacks viable cells, nerve regeneration and remodeling of extracellular matrix have been delayed [8]. The use of pre-degenerated nerve grafts having high matrix metalloproteinase (MMP) expression shows some potential as it degrades the inhibitory chondroitin sulphate and proteoglycans thereby retaining the ability to promote nerve regeneration even in the absence of cells [8,9].

**Figure 1:**
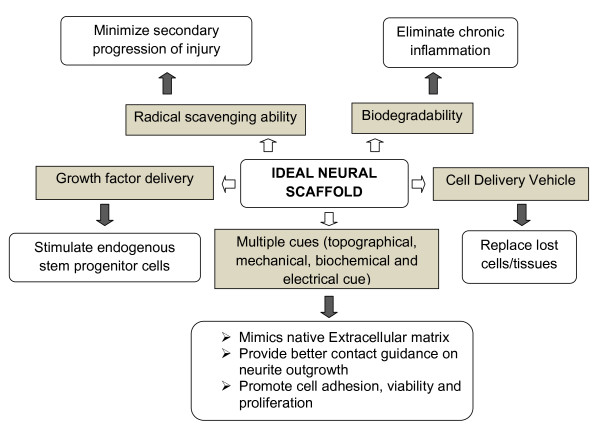
**Ideal properties of scaffold**.

Recent advances in nanotechnology [10] and tissue engineering [11,12] have been found to cover a broad range of applications in regenerative medicine and offer the most effective strategy to repair neural defects. The major determinant in all tissue engineering research is to regulate the cell behavior and tissue progression through the development and design of synthetic extracellular matrix analogues of novel biomaterials to support three-dimensional cell culture and tissue regeneration. Ideal properties of a scaffold for nerve regeneration are biocompatibility, less inflammatory, controlled biodegradability with non-toxic degradative products, porosity for vascularization and cell migration and three-dimensional matrices with appropriate mechanical properties to mimic the extracellular matrix [13-15]. Figure 1 shows the various characteristics desired for an ideal scaffold for neural regeneration.

Polymeric biomaterials are widely preferred as scaffolds for peripheral and central nerve regeneration both *in vitro *and *in vivo *[16-19]. There is a wide choice of polymers available with programmable biodegradability, non-toxic/non-inflammatory nature, mechanical properties similar to the tissue to be replaced, high porosity that promotes cell attachment and growth, economical and simple manufacturing processes along with a potential for chemical modification leading to increased interaction with normal tissue [20]. Several techniques such as nanofiber self-assembly, solvent casting and particulate leaching, gas foaming, emulsification/freeze-drying, liquid-liquid phase separation, electrospinning and computer aided design and manufacturing techniques have been employed to fabricate tissue engineering scaffolds with varying degrees of success [21-24]. Various attempts made towards repairing neural defects have been discussed in the following sections along with the rationale behind selection of a suitable scaffold material for successful neural tissue engineering.

### Response to Injury & Repair

Regeneration strategies for peripheral and central nervous system damage have not been very successful due to lack of knowledge about the mechanisms of nerve injury and repair [25]. Nerve cells have been found capable of easily bridging gaps of less than 6 mm [26]. Thus, regenerating nerves across larger gaps are medically challenging phenomenon since, injuries of less impact in peripheral nervous system (PNS) heal by formation of fibrin cable across the gap [4]. This eventually allows the Schwann cells to migrate from both the nerve ends, thereby orienting the bungner bands to promote neurite outgrowth [27]. Presently, researchers have investigated a suitable strategy to enhance the formation of Bungner bands and have identified microstructured biomaterial filaments provide a better topography, promoting bungner band formation even in the absence of biological factors [28]. Additionally, after injury, the protein synthesis and degradation machinery in axons play a major role in the initiation of growth cone formation [29]. However, the myelination of central nervous system (CNS) is distinct from that of peripheral nervous system (PNS). Astrocytes and oligodendrocytes are found in the CNS which also marks a key difference between CNS and PNS in their response to injury [16]. In the case of CNS, spontaneous regeneration is impossible due to its own inhibitory environment [16,25]. This includes glial scar formation and accumulation of myelin-associated inhibitors such as chondroitin sulphate proteoglycans [30,31]. Moreover, the primary injury in CNS expands further by damaging the nerve cells due to secretion of free radicals from the blood via the blood-brain barrier resulting in secondary injury which impedes the regeneration potency of these cells [32]. Many strategies have been attempted to improve the regenerative potency of neurons such as cell therapy, exogenous delivery of growth factors and tissue engineering approaches; each in turn restores the function with varying degrees of success.

### Regeneration Potential of Neural Cells

Guenard *et al. *have observed that the potential use of autologous Schwann cells that aid in CNS regeneration [33]. This novel approach modifies the environment of the CNS by transplanting Schwann cells which enhances axonal regeneration. This may be due to the fact that Schwann cells are the myelinating glial cells in the PNS and are known to play a key role in Wallerian degeneration and subsequent CNS regeneration [4,20,34,35]. Schwann cells promote neural regeneration and remyelination by secreting adhesion molecules L1 and neural cell adhesion molecule (N-CAM), extracellular molecules (collagen and laminin) and a number of tropic factors such as nerve growth factor (NGF), brain-derived neurotrophic factor (BDNF) and neurotrophin-3 (NT-3) [36]. Though the Schwann cells have regenerative potential, studies have shown the undesirable effects such as inhibition of Schwann cell migration into the CNS, delayed functional recovery and in certain cases, the Schwann cells may follow its original white matter pathway instead of the intended grey matter pathway [36].

The nearly impossible task of rebuilding the nervous system has since undergone a dramatic transformation with the discovery of stem cells [30,37]. The differentiation of neural stem cells to specific cell lineages has been controlled by constructing scaffolds of composite biomaterials that consist of extracellular matrix (ECM) components and growth factors [38]. The embryonic stem cell-derived oligodendrocytes have been recognized to myelinate axons in culture and to replace lost myelin in the injured adult CNS [39]. Human embryonic stem cell-derived oligodendrocyte progenitors when transplanted into injured spinal cords of rat proved to be a safe procedure, resulting in improved locomotor function [40]. The ideal cell culture conditions for embryonic stem cell proliferation and differentiation in fibrin-based scaffolds has been identified for neural tissue engineering applications [41].

Investigations on astrocytes have supported its role in the induction of neurogenesis from adult neural stem cells [42]. The directed growth of astrocytes on polymer substrates provides an innovative approach to promote controlled outgrowth and differentiation of neural stem cells [43]. Though the astrocytes contribute the cytotrophic effects in neural repair, at certain stages in response to injury it inhibits neurite outgrowth by releasing signals inhibiting neurite extension [44]. Researchers have tried to improve the cytotropic effects of astrocytes in regeneration by harnessing its cytotoxic effects [44].

In addition to these cells, olfactory ensheathing cells (OEC) [45] and trans-differentiated mesenchymal stem cells [46] have also been identified to promote axonal regeneration and functional recovery at the site of a spinal cord injury. The olfactory system promotes the axonal outgrowth into the CNS from the PNS due to the presence of both peripheral and central tissues [47]. Thus the phenotype of OEC is closer to the Schwann cells and has the properties of both Schwann cells and Astrocytes [36]. The transplantation of OEC enhances its migration [48] and secretion of extracellular molecules type IV collagen, tropic factors such as vascular endothelial growth factor (VEGF), NGF and BDNF [36]. The OEC are found to reduce the secondary neuron apoptosis and the degree of functional recovery after implantation of OEC is rapid as compared to Schwann cells [36,49].

Although the stem cell therapy seems to be promising, it is very hard to control the cell proliferation and differentiation into three-dimensional architectures of tissues [50]. Moreover, the use of stem cells to repair spinal cord has been reported to cause adverse side effects such as allodynia in unaffected forepaws of the rats [51]. Hence, it is a challenge to provide optimized conditions for controlled and adequate differentiation of transplanted stem cells in order to develop a safe stem cell-based therapy. Certain factors in stem cell-based therapy such as carcinogenicity and ethical concerns in the use of embryonic stem cells remain unanswered thereby limiting their use.

### Delivery of growth factors to promote regeneration

Growth factor signaling play a major role in tissue repair process. In addition to maximizing the intrinsic regenerative potency of endogenous progenitor cells, bioactive factors are also used to manipulate the differentiation and growth of exogenous stem cells [52]. The discovery of Nerve Growth Factor (NGF) has been more helpful to support ailing neurons by promoting nerve regeneration [53,54]. However, targeting and retaining the required concentration of these factors at the site of injury is quite complicated phenomenon. The nerve guidance channels provide a conduit for the diffusion of these growth factors and reduce the glial scar formation [16]. The sustained release of lipoplexes (complexes of liposomes and oligonucleotides) has shown promise to repair nerve injury by expressing neurotrophic factor [55,56]. Moreover, restoring the activity of such proteins is pretty challenging. Although Poly (D,L-lactic-*co*-glycolic acid) (PLGA) microspheres has been demonstrated as a potential carrier of growth factors, the main concern is the inactivation of proteins due to the release of acidic products. This problem can be overcome by using polyphosphoesters as a delivery vehicle [57]. Recently, Sun *et al. *targeted a collagen binding domain-nerve growth factor β (CBD-NGF β) to nerve extracellular matrix collagen to restore the peripheral nerve function in rat sciatic nerves [58]. They have confirmed the functional recovery by performing walking track, histological and electrophysiological analysis. These discoveries of regenerative capacity in adult central nervous system hold promise to neural victims for complete recovery [58]. Currently electrospun nanofibrous scaffold become a successful and safe delivery vehicle since it can simultaneously act as a scaffold and improve contact guidance while delivering the bioactive factors in controlled and sustained way [59].

### Biomaterials in Nerve Regeneration

Natural polymers (chitosan, chitin, collagen, gelatin, alginate), synthetic non-degradable polymers (silicone), synthetic biodegradable polymers such as PLGA, poly (ε-caprolactone (PCL), poly L-lactic acid (PLLA) and conducting polymers (polypyrrole, polyaniline) have been used in various nerve regeneration approaches. An ideal nerve conduit should be thin, flexible, porous, biocompatible, biodegradable, compliant, neuroinductive, neuroconductive and with appropriate surface and mechanical properties [14]. Although these biomaterials promise to fulfill some of the above stated criteria, they have some demerits which have to be overcome to meet the specific tissue engineering applications. For example, a scaffold made from non-degradable materials should be avoided to prevent the chronic inflammation and compression of nerve over time and therefore it is preferable to use biodegradable materials [26]. Even in biodegradable materials, surface erosion is desired over bulk erosion, since it permits the scaffolds to retain their structural stability for a longer time after implantation. Therefore, a surface eroding polymeric scaffold is expected to provide better contact guidance cues continuously for nerve regeneration. This fact is reinforced by a recent report on the use of poly (glycerol sebacate) (PGS) as a nerve guide material due to its surface erodible and elastomeric properties [60]. In general, researchers have attempted to improve the neural scaffold properties by several novel fabricating techniques such as polymer blending and electrospinning, incorporating nerve growth factors in the scaffold [61], and improving the wettability of the scaffold surface by surface modifications. Table 1[62-78] summarizes a list of biomaterials and techniques that have been used to promote nerve regeneration.

**Table 1:** Modified biomaterials attempted to promote nerve regeneration.

Biomaterials	Modification/Method of fabrication	Improved Properties	Ref
Star-Poly(ethylene glycol)	Incorporation of polysaccharide (Heparin)	Tunable physical and mechanical properties to adopt specific tissue requirements	[62]

Chitosan	Modified with (γ-glycidoxypropyltrimethoxysilane (GPTMS)	Mechanical strength	[63]

Poly(sialic acid)	Hydrogel modified with adsorbed poly-L-lysine or poly-L-ornithine or laminin or collagen	Mechano compatibility; Cell adhesive property	[64]

Poly(β-hydroxybutyrate)	Sheets impregnated with extracellular matrix molecules	Cell adhesion and proliferation	[65]

Poly(ε-caprolactone)	Electrospinning and Thermal fiber bonding	Mechanical strength	[66]

Poly(ε-caprolactone)	Aligned fibers by Electrospinning	Contact guidance	[67]

Poly(lactic-*co*-glycolic acid)	Modified immersion precipitation method	Selective permeability; Hydrophilicity	[68]

Poly(D, L-lactide-co-ε-caprolactone) [PDLLA/CL]	PPy coating substrate and PPy nanoparticle/PDLLA/CL composite	Electrical cue for multitude of cell functions	[69]

Chitosan	Polylysine-functionalised thermoresponsive chitosan hydrogel	Injectable scaffold; Mechano compatibility; Surface property (wettability, charge density)	[70]

Poly(ε-caprolactone)	Electrospinning (Polymer blending with collagen)	Biological property (schwann cell adhesion, migration and differentiation)	[71]

Collagen	Hydrogel crosslinked with YIGSR peptide modified dendrimers	Biological function (promote the growth of corneal epithelial cells and neurite outgrowth)	[72]

Poly(glycerol-sebacate)	Replica molding	Micropatterned substrates; Flexibility; Surface degradable; Strong contact guidance response	[73]

Poly(lactic-*co*-glycolic acid)	Microbraiding method	Flexibility; Porosity	[75]

Poly(D,L-lactide-*co*-glycolide)	Low pressure injection molding	Porosity; Longitudinally aligned channels; Mimics the geometry of native nerves	[76]

Poly(2-hydroxyethyl methacrylate)	Fiber templating technique	Oriented scaffold; Physical characteristics similar to soft tissue.	[77]

Poly(2-hydroxyethyl methacrylate)	Liquid-liquid centrifugal casting	Mechanical property similar to spinal cord	[78]

One of the most important properties needed for successful graft uptake by host tissues is mechanical stability and compatibility of the scaffolds. For nerve tissue engineering, the scaffold should be pliable, harmless to the surrounding tissues, resist structural collapse during implantation [79] which may lead to necrosis and inflammation. Many approaches have been attempted to improve the properties of common biomaterials to make them suitable for neural tissue engineering. A highly flexible PLGA scaffold was developed by microbraiding method to improve its flexibility and porosity [75]. Suitability of PLLA porous conduits fabricated by extrusion technique has been evaluated *in vivo *for scaffold applications [80-82]. Biomechanical properties of electrospun PCL scaffolds were improved by thermal treatment while retaining the structural stability, gross appearance, porosity and fiber diameter [66].

Polymer blending offers one of the most successful methods to develop a suitable scaffold with all preferred properties for specific tissue engineering applications [83,84]. For example, a polymer blend of the brittle-natured PLGA and a soft, elastic polymer such as polyurethane or poly (ethyleneglycol) (PEG) have been found to exhibit much greater elasticity than PLGA itself [85]. Chitosan-gelatin composite films show improved mechanical property and nerve cell affinity due to its softness and elastic properties [84]. Fabrication of biodegradable nerve guidance channels based on chitin/chitosan has been carried out for improvements in nerve tissue engineering [86]. An artificial nerve graft of chitosan/polyglycolic acid (PGA) blend has been used to bridge a 30 mm sciatic nerve defect in a large animal model [87]. Repairing long-term delayed peripheral nerve defects is clinically very challenging which includes number of various factors such as availability of surviving Schwann cells, worsening of growth permissive environment by disintegration of Schwann cell basement membrane [88]. Jiao *et al. *attempted to bridge the long term delayed defects of rat sciatic nerves using biodegradable chitosan-PGA graft and measured the functional recovery using histological and electrophysiological assays [88]. They have observed very few functional (regenerated) nerve fibers and poor growth support in delayed repair groups can improve the potency of chitosan/PGA grafts in delayed repair [88].

Cell adhesion property of a scaffold mainly depends on its surface characteristics such as charge density and wettability [66,89]. Most of the synthetic biodegradable materials are hydrophobic (PLGA, PCL, PHB etc.,) which limits their use as tissue engineering scaffolds. Thus surface modification either by coating of the surface with ECM proteins such as laminin, fibronectin, collagen or by incorporating specific adhesion peptide sequences like RGD and IKVAV, YIGSR can induce hydrophilicity which in turn improves the cell adhesion property of the scaffold. Currently the cell adhesion property of the materials such as methyl cellulose [90], alginate [91], poly (hydroxyethyl methacrylate) (PHEMA) [92], poly (hydroxybutyrate) (PHB) [65] has been improved by modifying their surface with specific peptide sequences.

Different approaches have been used to improve the biocompatibility. The performance of neural implants has been improved by using the layer-by-layer (LbL) technique [93]. A photochemical method has been employed to make PHEMA bound neural growth factor (NGF) more bioactive [94].

### Polymeric Scaffolds as Extracellular Matrix Analogues

Scaffolding is a temporary framework used to support cells in the construction or repair of tissue. Surface chemistry of scaffold materials is considered to be the most important parameter in tissue engineering [95]. The extracellular matrix (ECM) in biological systems holds the cells together and provides a medium for the cells to interact and migrate [96]. Thus it is desirable that the synthetic scaffold mimics the ECM in promoting cell adhesion, proliferation, and differentiation *in vitro *and *in vivo *[66,97]. Two dimensional tissue cultures as the name suggests offer only a monolayer of cells as opposed to the three-dimensional nature of tissue in organisms [98]. Hence it is an inadequate model for complex cellular interactions and is prone to hydrodynamic damage in bioreactors [98]. Three-dimensional tissue cultures have received much consideration than two dimensional cultures because of their superior hydrodynamic protection, higher surface area per unit volume, better cell-cell interaction and improved regeneration of the injured tissue [99]. Improved functional recovery and formation of neural networks to bridge the gap following spinal cord injury has been reported in the transplantation of stem cells on polymeric scaffolds than the transplantation of stem cell alone [30,100].

### Geometric Cues - Structure of Scaffolds

Well defined nanostructured topographical cues such as grooves, ridges, pores, nodes can influence cell-substrate interaction by promoting cell adhesion, migration, proliferation and differentiation to new tissue [101]. For example nanopatterned gratings on poly(methyl methacrylate) (PMMA) and poly(dimethylsiloxane) (PDMS) were used to induce alignment and elongation of smooth muscle cells [101]. There are different scaffold fabrication techniques such as solvent casting, particulate leaching, melt molding etc., to fabricate scaffolds of various geometries with the desired porosity and surface area for cell scaffolding [102]. Among the different forms of scaffold (nanofibers, sintered matrix, nanofoams, hydrogels, nanotubes, etc.,) hydrogels and nanofibers have been extensively investigated for use as a scaffold in neural regeneration.

In recent years, hydrogels have received considerable attention as a suitable scaffold material in neural tissue engineering [103,104]. Hydrogels provide appropriate chemical, mechanical and spatial microenvironment akin to the natural ECM to support the neurite extension for cell proliferation, differentiation and axon extension [95,105]. Moreover they are biocompatible and possess similar mechanical properties to soft tissue, low interfacial tension, and are good injectable scaffolds. A biocompatible polymeric hydrogel has been shown to induce reconstruction of the rat spinal cord after chronic compression-produced injury [55,106]. Neuroinductive and neuroconductive properties of a biocompatible heterogeneous poly [N-(2-hydroxypropyl) methacrylamide] (PHPMA) hydrogel have been used extensively to repair tissue defects in the central nervous system by promoting the formation of a tissue matrix and axonal growth [107]. Poly (2-hydroxyethyl methacrylate) (PHEMA) hydrogels have also proved to be useful in neural tissue engineering applications [77]. Cells get introduced easily into the liquid precursors of the gel due to its smaller mesh size. Production of cell-based hydrogel polymer constructs has been envisioned for tissue replacement in the central nervous system with combined physico-chemical properties such as biocompatibility, stability, porosity and hydrophilicity along with biological recognition such as expression of biospecific surface receptors and synthesis of bioactive molecules [108]. It has been reported that the use of polyethylene glycol (PEG) hydrogel as a cell carrier supported the neural precursor cell survival, expansion and differentiation in culture [109]. The evaluation of growth properties of Schwann cells on chitosan proved its biocompatibility [110]. Implantation of three-dimensional polymeric hydrogel into the site of injury has been attempted to enhance the axonal recovery [111]. Three-dimensional polylysine-functionalized polysaccharide hydrogel system promises to be a good scaffolding material for neural tissue engineering [70,112]. Three-dimensional peptide channels within hyaluronan (HA) hydrogel matrix modified with S-2-nitribenzyl cysteine (HA-SNBC) is expected to serve as a temporary scaffold for guided axonal regeneration *in vivo *[113]. Among the various polymeric hydrogel tubes that have been designed and studied for their suitability as neural scaffolds, reinforced coil tube expressed excellent mechanical properties equivalent to soft tissues and they supported neural outgrowth. Also it was observed that the conducting velocity of the construct exactly matched that of native axons [114].

### Nanofiber Properties & Electrospinning Technology

**Figure 2:**
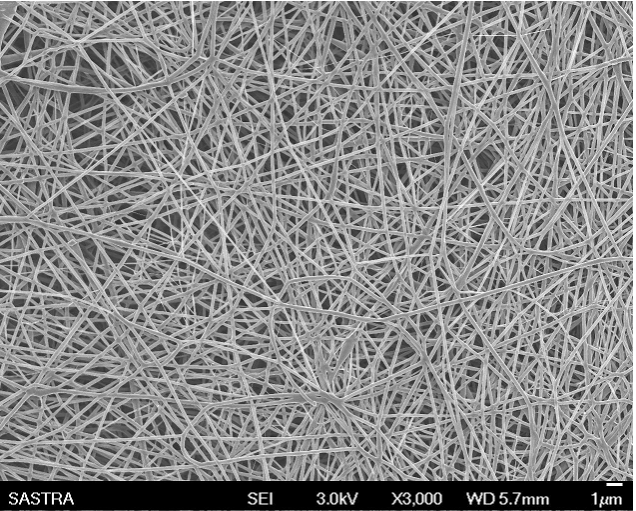
**Electrospun PLGA-PANi nanofibers**.

Nanofiber seems to be most promising substrate in tissue engineering applications due to its resemblance to native extracellular matrix [67,115,116]. Nanofiber is a broad phrase generally referring to fibrous structures with a diameter less than 1 micron. Figure 2 shows the scanning electron micrograph of electrospun PLGA-Polyaniline nanofibers with smooth defect-free morphology for neural tissue engineering. Extraordinary mechanical strength and high surface area to volume ratio makes the nanofiber more suitable for neural tissue engineering [10,117]. Porous polymeric nanofibrous scaffold using biodegradable poly (L-lactic acid) (PLLA) fabricated by liquid-liquid phase separation method resembles ECM of natural collagen to support neuron differentiation and neurite outgrowth [22]. However, it is very difficult to maintain the fiber diameter and alignment using this technique. Likewise, various techniques have been reported to develop nanofibers namely, template synthesis, phase separation, self-assembly, drawing and electrospinning [115]. Among these techniques, electrospinning offers more advantages due to its ease of fabrication. Nanofibrous conduit comprising poly (D,L-lactide-*co*-glycolide) and poly(ε-caprolactone) (PCL/PLGA) were found to promote nerve regeneration across 10 mm nerve gap in rat sciatic nerve [118]. PLGA random nano and microfibers, aligned microfibers and films were investigated for C17.2 neural stem cell culture and recognized the differentiation of neurons along the fiber direction [119].

PLLA nanofibrous scaffolds were developed via electrospinning and found to support neural stem cell (NSC) adhesion, outgrowth and differentiation [21]. Suitability of aligned electrospun PLLA nanofibers compared with random nanofibers was evaluated for neural tissue engineering in terms of their fiber alignment and dimension [21]. The aligned nanofibers were found to support the orientation of cells and improve the neurite outgrowth and contact guidance [21,67]. Based on the experimental results, this study recommended the aligned PLLA nanofibrous scaffold as a potential cell carrier in neural tissue engineering [21,119]. The parameters such as viscosity, conductivity, surface tension of polymer solution, applied electric potential, flow rate, and distance between the electrodes are to be optimized while carrying on the electrospinning process [120]. It is also observed that the orientation of fiber became disordered at the top layer of the electrospun mesh when the collecting time was longer than thirty minutes due to the residual charges on the collecting fibers [21]. The desirable properties of electrospun nanofiber scaffolds seem to offer a promising alternative towards the treatment of neural defects. Aligned electrospun collagen/PCL fibers supports cell proliferation, glial migration, orientation of neurite outgrowth, suggested its suitability as nerve implants [71]. Aligned electrospun PCL fibers have been found to up regulate specific genes such as P0 and down regulate NCAM - 1 on cultured Schwann cells thereby promoting the Schwann cell maturation [67]. Significantly higher Schwann cell migration and neurite outgrowth was observed on uniaxially aligned fibers of poly (acrylonitrile-*co*-methylacrylate) (PAN-MA) developed by electrospinning than on random fibers [121].

### Other Approaches

Fabrication of a multi-channel scaffold using injection molding with solvent evaporation technique has been demonstrated to promote spinal cord axon regeneration [122]. A new facile method named 'fiber stimulating technique' used to fabricate oriented PHEMA scaffolds successfully for neural tissue engineering, promises to be more effective and reproducible [77]. Melt compression and melt extrusion are also considered to be viable techniques to prepare nerve guides [14]. Innovative fabrication techniques such as wire mesh method and mandrel adhesion method are used to prepare multi-channel biodegradable nerve guides without the requirement of complex instrumentation, acidic conditions or exposure to extreme temperatures [123]. Designing of biodegradable PLGA hollow fiber by wet phase inversion technique has been attempted for the development of nerve tract guidance conduit [13]. Micropatterning is a novel patterning technique for biodegradable polymers and is reported to enhance peripheral nerve regeneration by controlling the alignment of Schwann cells [124-126]. Fabrication techniques continue to evolve novel routes to provide the most suitable nanostructure topography for adequate neural growth.

### Electrical Cues

**Figure 3:**
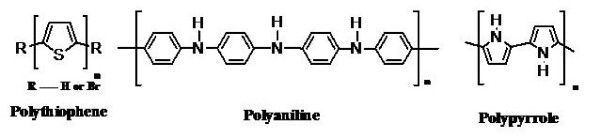
**Chemical structure of conducting polymers**.

Human body responds to electrical fields and the key component of neural communication in the body is the action potential generated at the synapse. This implies that an ideal neural scaffold should also possess electrical conductivity to promote neurite outgrowth and thereby enhance nerve regeneration in culture. The use of electrically conducting polymers in biomedical applications has become more attractive due to its tailor-made specificities [127]. Polypyrrole (Ppy), a well-known conducting polymer used in biomedical applications has been found to enhance the nerve regeneration by electrical stimulation [128,129]. Moreover, the antioxidant property of polypyrrole and polyaniline makes them more attractive substrates for tissue engineering applications [130,131] as they could scavenge any free radicals at the site of injury minimizing scar formation which is a bane of neural regeneration. The structures of polythiophene, polyaniline and polypyrrole are shown in figure 3.

A comparative study was made on the efficacy of two different laminin fragments p20 and p31 as dopants in conducting polypyrrole surfaces for *in vitro *growth of neurons. The results indicated that p20 as dopant supported the highest neuronal density than p31 dopant [132]. Conducting Ppy/PDLLA/PCL composites have been implanted to bridge the gap of 8 mm in rat sciatic nerves and shown to promote the nerve cell proliferation and axon regeneration using electrical cues [69]. The rats were gradually recovered the mobility in operated limb over the period of 2 months [69]. Moreover, the immunohistological analysis and transmission electron microscopy of harvested implants demonstrated the presence of newly formed myelinated axons and Schwann cells similar to that of native nerve [69]. Recently the cell adhesion property of polypyrrole has been improved by chemical conjugation of a functionalized carboxylic acid group with RGD peptide [133]. The suitability of poly ethylene dioxythiophene (PEDOT) as a biomaterial was evaluated by studying the adhesion and proliferation of epithelial cells and was demonstrated that the electroactive substrate favors cell adhesion [134]. Though the mechanism in which electrical stimulation promotes nerve regeneration is not clearly understood, several possible hypothesis have been postulated to elucidate their role in nerve regeneration. Some of the plausible reasons include electrophoretic redistribution of cell surface receptors, activate growth controlling transport process and altered adsorption of adhesive proteins [135]. The later hypothesis has been proved by stimulating the adsorption of fibronectin (ECM adhesive glycoprotein) from serum to polypyrrole surface via electrical stimulation, which subsequently facilitate the neuronal attachment and neurite outgrowth [128].

However, these conducting polymers are non-biodegradable and questions on their safety in biological systems have delayed their wide-spread use in neural conduits.

### Future Prospective

Many Challenges still remain unwrapped. Though the researchers have found different strategies to achieve the functional recovery to some extent, regaining the maximal or full function remains unexplored. There are some issues listed below have to be addressed in future; (1) the first inescapable conclusion arising over various reports on nerve tissue engineering by super positioning of all these approaches is crucial for promoting the neural regeneration on multiple levels (2) the probable hazards of long term usage of such novel biomaterials on human health yet to be revealed (3) the need for novel Biomaterials and approaches has to be established in order to treat the delayed nerve injuries in patients who have neurological disorders.

## Conclusion

An ideal nerve conduit requires a suitable porous, biocompatible, biodegradable, neuroconductive, neuroinductive, infection resistant, compliant three-dimensional biomaterial scaffolds. The engineered construct should also mimic the ECM architecture and porosity, desirable for cell attachment and other vital functions. Biomedical nanotechnology, electrospinning techniques and tissue engineering methods give us exciting insights to the design of a scaffold with good electrical, mechanical, biological properties and compliance match closely resembling the native ECM. Such scaffolds can also avoid infections, multiple surgeries and additional cost to the patient. An array of methods has been used for polymer scaffold preparation but electrospinning scores high due to its ease of operation, better control of fiber properties and desirable results. Lot of synthetic biodegradable polymers has been used till date but at the same time suffer from the demerits of release of acidic degradation products, hydrophobicity, poor processability and loss of mechanical properties. Also they support elongation and partial collapse of nerves. Hydrogels mimic soft tissue properties but are very difficult to sterilize and handle due to their fragile nature. A new strategy using polymers like polypyrrole, polyaniline etc., having conducting properties are being investigated for neural tissue engineering to stimulate neurite extension. However, the biocompatibility of these polymers has not been conclusively proved till now. It is seen that though different classes of biomaterials are available, no single material is enough to improve the scaffold properties for nerve regeneration. Hence, the major challenge in developing a scaffold lies primarily in the choice of a blend of biomaterials with the correct combination of properties. The field is still wide open to design the most appropriate polymer scaffold with all the vital conditions and properties for effective neural applications *in vivo*.

## Competing interests

The authors declare that they have no competing interests.

## Authors' contributions

All authors have equal contribution in the preparation of the manuscript.
